# Supplementary material for: The Systems Biology Research Tool: evolvable open-source software
Source: BMC Syst Biol. 2008 Jun 29;2:55. doi: 10.1186/1752-0509-2-55 (PMC2446383; doi:10.1186/1752-0509-2-55)
Supplement: Additional file 1 — SBRT Archive. An archive of the current version of the Systems Biology Research Tool. [file 1752-0509-2-55-S1.zip › sbrt-1.4.0/doc/developers_guide/api/sbrt/shell/text/Parser.html]

Parser


|  |  |  |
| --- | --- | --- |
| **Overview** \| **Package** \| **Class** \| **Use** \| **Tree** \| **Deprecated** \| **Index** \| **Help** | |  |
| **PREV CLASS**   **NEXT CLASS** | **FRAMES**    **NO FRAMES**     **All Classes** |  |
| SUMMARY: NESTED \| FIELD \| CONSTR \| METHOD | DETAIL: FIELD \| CONSTR \| METHOD |  |


---


## sbrt.shell.text Interface Parser<T>

**Type Parameters:**: `T` - the type of object this format applies to.

**All Known Subinterfaces:**: BooleanFormat, CatalystFormat, CatalystListFormat, CatalystSetFormat, ChemSpeciesFormat, CoeffFormat, CollectionFormat<E>, DirEdgeFormat<N>, DoubleFormat, FbaOptHeaderParser, IndexPairFormat<R,C>, IntegerFormat, IntervalFormat, LinearCombFormat<V>, LinearCombTermFormat<V>, LinkedHashSetFormat<E>, ListFormat<E>, LongFormat, MathExprFormat<E,V>, MatrixElementFormat<R,C,E>, PercentFormat, RxnNameExprFormat<E,S>, RxnNameFormat<T>, RxnNameListFormat, RxnNameOrExprFormat, RxnNameSetFormat, SimpleFormat<T>

**All Known Implementing Classes:**: AbstractLinkedHashSetFormat, AbstractListFormat, AmpersandSetFormat, BoolRelFormatV1, BoundedIntervalFormatV1, CatalystFormatV1, CatalystFormatV2, CatalystListFormatV1, CatalystSetFormatV1, CatalystVerifier, ChemSpeciesFormatV1, ChemSpeciesVerifier, CoeffFormatV1, CoeffFormatV2, DirEdgeFormatV1, DoubleFormatV1, DoubleFormatV2, FbaOptHeaderParserV1, FileFormatFormat, IndexPairFormatV1, IntegerFormatV1, IntervalFormatV1, IrrevRxnFormatV1, IrrevRxnFormatV1, IrrevRxnFormatV2, LinCombTermFormatV1, LinCombTermFormatV2, LinearCombFormatV1, LinearCombFormatV2, LongFormatV1, MatrixElementFormatV1, NonEmptyStringFormat, PercentFormatVersion1, PipeListFormat, PipeSetFormat, RevRxnFormatV1, RevRxnFormatV2, RxnNameExprFormatV1, RxnNameFormatV1, RxnNameListFormatV1, RxnNameOrExprFormatV1, RxnNameSetFormatV1, RxnNameVerifier, SenseFormat, SimpleListFormat, SimpleSetFormat, StringFormat, TabListFormat, TabSetFormat, TrueFalseFormat, WhitespaceListFormat, WhitespaceSetFormat, YesNoFormat

---

``` public interface Parser<T> ```

This interface is used to represent *parsers*.
A parser is used to "convert" a single formatted
string into a single object.

**Author:**
:   This interface was written and documented by
    Jeremiah Wright while in the Wagner lab.

---

| **Method Summary** | |
| --- | --- |
| `T` | `parse(java.lang.String source)`             Parses the provided string and returns its corresponding object. |

**Method Detail**

### parse

```
T parse(java.lang.String source)
```

:   Parses the provided string and returns its
    corresponding object.

    :   **Parameters:**: `source` - the string to be parsed. **Returns:**: the object corresponding to the provided string.


---


|  |  |  |
| --- | --- | --- |
| **Overview** \| **Package** \| **Class** \| **Use** \| **Tree** \| **Deprecated** \| **Index** \| **Help** | |  |
| **PREV CLASS**   **NEXT CLASS** | **FRAMES**    **NO FRAMES**     **All Classes** |  |
| SUMMARY: NESTED \| FIELD \| CONSTR \| METHOD | DETAIL: FIELD \| CONSTR \| METHOD |  |


---
